# A community-based strategy to eliminate hepatitis C among people who inject drugs in Vietnam

**DOI:** 10.1016/j.lanwpc.2023.100801

**Published:** 2023-05-27

**Authors:** Nicolas Nagot, Nguyen Thanh Binh, Tran Thi Hong, Vu Hai Vinh, Catherine Quillet, Roselyne Vallo, Duong Thi Huong, Khuat Thi Hai Oanh, Nham Thi Tuyet Thanh, Delphine Rapoud, Bach Thi Nhu Quynh, Duc Quang Nguyen, Jonathan Feelemyer, Laurent Michel, Peter Vickerman, Hannah Fraser, Laurence Weiss, Maud Lemoine, Karine Lacombe, Don Des Jarlais, Pham Minh Khue, Jean Pierre Moles, Didier Laureillard, DRIVE-C study group, Didier Laureillard, Nicolas Nagot, Don Des Jarlais, Jonathan Feelemyer, Catherine Quillet, Roselyne Vallo, Jean-Pierre Moles, Laurence Weiss, Maud Lemoine, Laurent Michel, Pham Minh Khue, Delphine Rapoud, Nguyen Thanh Binh, Tran Thi Hong, Nguyen Thi Thanh Hang, Phung Quang Hai, Vu Thi Thom, Cap Minh Duc, Le Thi Thuy Linh, Bach Thi Nhu Quynh, Le Thuy Linh, Nguyen Thi hong, Vu Thi Thu Trang, Vu Hai Vinh, Nguyen Thi Thanh Huong, Hoang Thi Thia, Nguyen Thi Van Anh, Vu Thi Sinh, Doan Thi Hai Binh, Nguyen Van Luc, Vu Thi Thu Ha, Do Thi Nhung, Bui Minh Khoi, Bui Thi Thien, La Thi Vu Quang, Le Huu Toi, Hoang Thi Luong, Pham Thi Thanh Phuong, Pham Thi Lieu, Pham Thi Thanh Mai, Trinh Thi Hoa, Bui Thi Thu Trang, Nguyen Thi Thu Ha, Nguyen Thi Xuyen, Trinh Thi Huong, Nguyen Thi Minh, Phung Thi Thuy, Tran Thi Duyen, Vu Thi Que, Nguyen Duc Dung, Nguyen Duc Tuan, Nguyen Hoang Long, Nguyen Manh Hung, Vu Van Tu, Nguyen Thanh Kien, Pham Thi Thu Hong, Truong Thi Cuc, Nguyen Minh Quan, Nguyen Thi Lan, Nguyen Thi Va, Nguyen The Manh, Doan Van Cuong, Pham Van Hoan, Cao Thi Kim Giang, Ha Quang Hiep, Ngo Trong Ninh, Nguyen Anh Quan, Vu Thi Bich Hop, Nguyen Thi Thu, Nguyen Thi Sau, Hoang Van Tuan, Nguyen Huu Nhan, Nguyen Quoc Tuan, Le Tuan Long, Vu Minh Son, Tran Viet Hoc, Nguyen Van Dinh, Nguyen Hoang, Pham Thi Hung, Trinh Thai Binh, Le Thi Hang, Hoang Dang Quan, Nguyen Thi Thanh, Le Thi Loan, La Cao Cuong, Tran Van Ha, Tran Van Thao, Nguyen Xuan Truong, Nguyen Duc Hanh, Nguyen Thi Loan, Tran Lam Tung, Tran Thi Lien, Khuat Thi Hai Oanh, Nham Thi Tuyet Thanh, Bui Thi Thuy Linh, Ngo Thi Dien, Peter Vickerman, Hannah Fraser, Josephine Walker, Adam Trickey

**Affiliations:** a Pathogenesis and Control of Chronic and Emerging Infections, Université de Montpellier, Inserm, Etablissement Français du Sang, Montpellier, France; b Faculty of Public Health, Hai Phong University of Medicine and Pharmacy, Hai Phong, Vietnam; c Infectious and Tropical Diseases Department, Viet Tiep Hospital, Hai Phong, Vietnam; d Center for Supporting Community Development Initiatives, Hanoi, Vietnam; e Department of Molecular Biology, Hai Phong University of Medicine and Pharmacy, Hai Phong, Vietnam; f School of Global Public Health, New York University, New York, USA; g CESP Inserm UMRS 1018, Paris Saclay University, Pierre Nicole Center, French Red Cross, Paris, France; h Population Health Sciences, Bristol Medical School, University of Bristol, Bristol, UK; i Université de Paris Cité, Department of Clinical Immunology, Hôpital Hôtel Dieu AP-HP, Paris, France; j Liver Unit, Division of Digestive Diseases, Department of Metabolism, Digestion and Reproduction, Imperial College London, London, UK; k Sorbonne Université Inserm IPLESP, Hôpital St Antoine AP-HP, Paris, France; l Infectious and Tropical Diseases Department, Caremeau University Hospital, Nîmes, France

**Keywords:** Hepatitis C, People who inject drugs, Respondent-driven sampling, Community-based organisations

## Abstract

**Background:**

Towards hepatitis C elimination among people who inject drugs (PWID), we assessed the effectiveness of a strategy consisting of a community-based respondent-driven sampling (RDS) as wide screening, a simplified and integrated hospital-based care, and prevention of reinfection supported by community-based organisations (CBO), in Hai Phong, Vietnam.

**Methods:**

Adults who injected heroin were enrolled in a RDS survey implemented in two CBO premises. Rapid HIV and HCV tests were done on site, and blood was taken for HCV RNA testing. Those with detectable HCV RNA were referred with CBO support to three public hospitals for 12-week sofosbuvir/daclatasvir, plus ribavirin for patients with cirrhosis. Participants were followed-up 12 weeks post-treatment (SVR12) and 48 weeks after enrolment. The primary endpoint was the rate of undetectable HCV RNA participants at 48 weeks.

**Findings:**

Among the 1444 RDS survey participants, 875 had hepatitis C. Their median age was 41 years (IQR 36–47), 96% were males, 36% were HIV-coinfected. Overall, 686 (78.4%) started sofosbuvir/daclatasvirs, and 629 of the 647 (97.2%) patients tested at SVR12 were cured. At week 48 (581/608) 95.6% had undetectable HCV RNA, representing 66.4% of all PWID identified with hepatitis C. The reinfection rate after SVR12 was 4/100 person-years (95% CI: 2–7).

**Interpretation:**

Our strategy, involving CBO and addressing all steps from wide HCV screening to prevention of reinfection, stands as a promising approach to eliminate HCV among PWID in low and middle-income countries.

**Funding:**

France ANRS|MIE (#ANRS12380). The RDS survey was implemented with grants from the 10.13039/100000026NIDA (#R01DA041978) and ANRS|MIE (#ANRS12353).

**Research in context**

**Evidence before this study**

We searched PubMed from 2015 to September 1st, 2022 to identify interventions among PWID that aimed at controlling HCV infections, including referral to care, HCV care outcomes and prevention of reinfection. We used combinations of the following terms: “PWID” “HCV or Hepatitis C” “elimination” “intervention” “treatment or antiviral” “screening” “referral to care” “reinfection” “low-middle income countries”. We retrieved 290 articles and cross-checked their references.

We found some interventional studies in low-middle income countries (LMIC) addressing one or several steps of the hepatitis C (HCV) screening and care cascade, but none encompassing the whole continuum from wide screening to HCV care and prevention of reinfection. Among the study reports retrieved, for each step of the cascade, the success rate was variable, and the overall level of evidence was poor, with either low number of participants or imprecisions in the strategy used or in reporting. Some health facility-based studies reported reasonable cure rate among people who inject drugs (PWID), although on low number of participants, while the largest study from India found a cure rate of 40% only among those treated. The respondent-driven sampling (RDS) approach has been deemed as a good strategy to identify HCV-infected PWID. A pilot study from Baltimore, USA, reported the value of peers and their network for the recruitment and referral to care of PWID with HCV. A meta-analysis provided good estimates of the post-treatment reinfection rate among PWID from upper-middle income countries.

**Added value of this study**

The HIV or MOUD (medication for opioid use disorders) facility-based approach for HCV testing and care used by previous studies let aside the large proportion of PWID not engaged in care, restricting the intervention coverage and therefore the chances to achieve elimination. Our strategy, including HCV screening based on RDS survey conducted by community-based organisations, allowed recruiting and testing large numbers of PWID from the community, in a short time frame. About half of PWID identified with HCV were not engaged in MOUD nor HIV care. This community network-based screening strategy, together with CBO support for HCV referral, care support and harm reduction counselling using a case-management approach, and an integrated, decentralised model of care, achieved very high rate of HCV cure. Overall, 66.4% of PWID identified with HCV at the RDS survey have been cured and remained free of HCV at one year. This strategy and these findings, unique in LMIC, encompassing the wide spectrum of HCV care from testing to reinfection after cure, have the potential to eliminate HCV in this high-risk group. We previously showed that the repetition of RDS surveys in the same city could allow recruiting up to two thirds of the whole target population.

Our findings also shed light on the crucial role of CBO at all steps of the intervention, from hosting and implementing the RDS, supporting PWID for both their referral to HCV care and treatment adherence, to preventing reinfection through harm reduction.

**Implications of all the available evidence**

The findings from our study, implemented in a 2-million inhabitant city in Vietnam, can be generalised to other LMIC settings, but also to high-income countries such as France where it was successfully replicated (ICONE study). The available evidence will inform modelling exercises to confirm that HCV elimination is possible using our strategy, would community-based RDS surveys be repeated to cover a large proportion of the local PWID population. Our results strongly suggest that CBO, or peers, currently involved in harm reductions in LMIC should be deeply involved in all initiatives of HCV elimination among PWID. Finally, potential concerns or hesitation for treating HCV among PWID due to presumed suboptimal adherence or high reinfection rate should no longer stand in LMIC.

## Introduction

In 2016, the WHO set targets for an ambitious goal of HCV elimination by 2030, through 90% testing and 80% treatment coverage.^1^ The emergence of generic direct antiviral agents (DAA), and the recent major cost reduction for Low-Middle Income Countries (LMIC), makes this worldwide goal attainable in a near future. One of the conditions to achieve this goal is to control hepatitis C virus (HCV) infection among people who inject drugs (PWID), the group carrying the highest hepatitis C burden.^2^^,^^3^

Worldwide, PWID are highly stigmatised with poor access to health services. Nevertheless, in high-income countries, PWID treated with DAA can achieve high rates of HCV cure, similar to those reported in the general population.^4^^,^^5^ In LMIC, where social, cultural, PWID patterns and levels of care much differ from those in high-income countries, the upcoming availability of DAA prompts the urgent need for the elaboration of strategies to control HCV among PWID.

HCV control programs targeting PWID first focused on PWID already engaged in care or harm reduction services, by setting-up HCV screening and care within dedicated facilities. In LMIC, such initiatives have been tested with limited success in addiction centres with about 50% cure rate in India among PWID who initiated DAA,^6^ or within HIV clinics with 62% HCV antibody testing rate.^7^ A similar approach has also been tested in community-based structures such as drop-in or needle and syringe program (NSP) centres,^8^^,^^9^ with more success, although the testing and reinfection rates were not included in the evaluation.

Nevertheless, HCV elimination requires a very high coverage of prevention, testing and care, which needs to extend beyond the proportion of PWID engaged in care^10^ and harm reduction services. In 2019, the Lancet Gastroenterology & Hepatology Commission^3^ as well as the recent updated WHO guidelines for the management of hepatitis C^11^ identified key priority areas to accelerate the elimination of viral hepatitis*.* They highlighted the need to develop and test models of screening and care, including innovative simplified care pathways integrated within existing services, decentralisation and task-shifting.^11^ Similar research priorities were delineated for PWID more specifically, emphasising the need to engage the community in the HCV elimination framework.^12^^,^^13^

Respondent-Driven Sampling is very efficient for reaching rapidly large numbers of PWID within a community.^14^ This sampling strategy was designed for epidemiological purposes, but it could also be used to screen large number of PWID. In Athens, the repetition of RDS surveys, together with linkage to care, was key to rapidly control an emerging HIV epidemic among PWID after the 2008 economic crisis.^15^ Repeating RDS surveys allowed reaching a high coverage (>80%) of the PWID population in the city. In India, RDS surveys implemented in several cities enabled to identify large numbers of PWID with unknown HIV and HCV infection,^16^ and a pilot study highlighted the potential of peers and network-based recruitment for improved linkage to care.^17^

Peers, through community-based organisations (CBO) can host and conduct RDS surveys, they can provide harm reduction counselling, assistance for linkage to care after testing, and support for drug adherence and follow-up.^18^ Within the DRugs & Infections in ViEtnam (DRIVE) project, which undertook annual RDS surveys to monitor the HIV epidemic among PWID in Hai Phong, Vietnam, we showed that CBO have been instrumental in ending the HIV epidemic among the PWID population despite high prevalence of HIV (25–30%).^19^

Lastly, the integration of CBO members in the HCV care team of the local clinics may reassure PWID and overcome potential stigma. CBO members could also help develop individualised modes of drug supply, and give support on drug adherence and follow-up.

In Vietnam, despite high medication for opioid use disorders (MOUD) coverage (30–40%) and low rates of needle and syringe sharing (<5%) due to easy access to sterile equipment, recent data report high levels of HCV seroprevalence (>70%)^20^ and incidence (19/100 person-years)^21^ among PWID. This alarming situation calls for an urgent need of additional strategies to reduce the burden of HCV among PWID in LMIC. Although DAA are technically speaking available in the country, they remain inaccessible to PWID due to their high cost. The government has recently introduced the partial reimbursement (50%) of HCV treatment in the national health insurance scheme, which has not increased their accessibility for PWID. In a view of HCV elimination, we took the opportunity of the DRIVE project to assess the efficiency of a community-based strategy aiming at screening and treating hepatitis C, and preventing HCV reinfection among PWID in Vietnam.

## Methods

### Study design

We designed an interventional study in Hai Phong, Northern Vietnam, a city with approximately two million inhabitants. The RDS-based recruitment was implemented in two CBO premises located at opposite sides of the city. Then, participants identified with hepatitis C were referred to one of three study clinics located in public hospitals for subsequent study visits. The study was approved by ethics and regulatory authorities of Vietnam (#19/HPUMPRB). The study protocol is detailed elsewhere.^22^

### Participants

Adult PWID aged 18 years or above, currently injecting, defined by self-report of drug injection, confirmed by the detection of heroin in urine and presence of recent skin marks, were eligible for the RDS survey. Heroin was in effect the only drug injected in the city at the time of the study. CBO identified 24 seeds within the community to launch the RDS, chosen for their important social network, their diversity of characteristics, irrespective of their HCV status.

### Procedures

After informed consent, eligible PWID with a valid coupon of participation were tested for the presence of heroin in urine. After an interview performed by trained CBO members on socio-demographic characteristics, drug use behaviours and care background, blood was sampled on site by a nurse for HCV and HIV serology using rapid tests (SD Bioline® HCV; SD Bioline® HIV 1/2 3.0, Standard Diagnostic Inc., South Korea). When the HCV rapid test was positive, participant blood samples were sent to the Hai Phong University of Medicine and Pharmacy laboratory for hepatitis C confirmation using Xpert® HCV Viral Load (Cepheid, California, USA) (hepatitis C defined by HCV RNA >10 IU/mL). Participants were compensated for their time and transportation (200,000 VND, i.e., 8€), and were given three numbered coupons to recruit other participants. Participants who distributed coupons that were returned by new participants received 50,000 VND (2€) per coupon for their efforts.

### Description of the intervention

#### HCV care

In order to improve access to health care services,^23^ the involvement of peers through CBO is pivotal to building trust with PWID. RDS survey participants came back to CBO premises one week later to receive their hepatitis C result and coupons incentives, along with 4–8 euros for transportation costs. Those with detectable HCV RNA were given an appointment by CBO members to attend one of the three study HCV clinics. CBO members called participants who missed this day 7 visit, and met with them in town when necessary.

At the HCV clinic, after informed consent for HCV treatment and follow-up, the clinic-based CBO member updated contact details and the RDS questionnaire on drug use behaviours. Then, a pre-therapeutic assessment was undertaken, consisting of clinical and blood examinations, liver fibrosis assessment using either the AST to Platelet Ratio Index score (APRI) and then Fibroscan (Echosens®, Paris, France) for those with an APRI >1, or only Fibroscan (in one of three clinics). Cirrhosis was defined as a Fibroscan value >12.5 kPa. Methadone and/or ART initiation (for HIV-positive PWID) in local clinics were encouraged with assistance from the CBO.

During a second visit one week later (after receiving blood examination results), participants were initiated on to a generic DAA regimen 19163126 comprised of sofosbuvir (SOF) 400 mg plus daclatasvir (DCV) 60 mg (SOF/DCV60) for 12 weeks if they had no contra-indications (e.g., pregnancy/breastfeeding, creatinine clearance <30 mL/min, decompensated cirrhosis, suspected hepatocarcinoma, other contra-indication to either DAA). Ribavirin (RBV) was added for cirrhotic patients (or the regimen extended to 24 weeks in case of ribavirin contra-indication), and the 60 mg dose of DCV was increased to 90 mg (SOF/DCV90) in patients receiving nevirapine or efavirenz. By default, DAA were supplied from the study clinic at treatment initiation, weeks 2, 4, 8, from the hospital pharmacy, plus weeks 12, 16, and 20 for those receiving a 24-week regimen. Participants were then followed at the HCV clinic 12 weeks after the end of treatment and at 48 weeks after treatment initiation visit. During these visits, a blood sample was taken for HCV RNA detection and CBO members interviewed participants on HCV risks and drug use behaviours since the previous visit. All HCV care above were free of charge for participants, and their transportation costs were reimbursed at each visit. The physician together with CBO members tailored this drug supply scheme to the participant characteristics, when necessary, by adding drug supply visits.

#### Prevention of HCV reinfection

At each drug supply visit, CBO members provided counselling on treatment adherence and routine harm reduction. During post-treatment visits, the prevention of HCV reinfection was reinforced through group harm reduction sessions using dedicated tools designed by CBOs, such as flipchart, including the risk of sharing water vials or novocaine for heroin dilution. Individual sessions were also carried out using a case-management approach, with provision of harm reduction clean equipment. When deemed necessary by the CBO member, additional counselling sessions were proposed at the CBO site.

### Study end points

The primary outcome was the strategy success, encompassing both hepatitis C cure and prevention of reinfection, defined by HCV RNA <10 IU/mL at 48 weeks. The secondary outcomes included sustained virological response at 12 weeks post-treatment (SVR12), reinfection rates at 48 weeks among those cured at SVR12, safety, and drug adherence.

### Data collection and analyses

All research data were recorded using an eCRF solution. Regular internal data monitoring and consistency checks were carried out to improve data quality.

We expected that 90% of participants with hepatitis C would be referred, 90% of those referred would initiate DAA and 90% of those who initiated DAA would be cured (SVR12), giving a 73% cure rate. At 48 weeks, we estimated 70% of RDS survey participants would still have undetectable HCV RNA. The recruitment of 900 participants with hepatitis C allowed estimating this cure rate at 48 weeks with a 38,195% confidence interval ranging from 67.0% to 73.0%. Given an estimated 70% HCV seroprevalence rate and 15% HCV spontaneous clearance, we aimed at enrolling 1500 PWID in the RDS survey. Study endpoints were estimated using proportions and their 95% confidence intervals. For the main analysis, we considered that participants with detectable HCV RNA at 12 weeks post-treatment, or who did not reach the week 48 visit, for any reason, were strategy failures.

The reinfection rate was calculated among those with SVR12 and who attended the week 48 visit. The number of re-infections was divided by the total number of person-years of follow-up, censoring patients with reinfection at the mid-point between their SVR12 and week 48 visit date. Poisson 95% confidence intervals were calculated. The clinical outcomes were also estimated among participants not engaged in HIV or substitution care at the RDS survey. We then identified the factors associated with the absence of treatment initiation, as we expected the main loss of participants would occur between the HCV result announcement and the clinic attendance. The multivariable logistic regression model was developed with variables associated with the absence of treatment initiation (p < 0.15) in univariate analyses, using a backward procedure taking into account the likelihood-ratio test statistic and AIC criteria. Statistical analyses were carried out using SAS/STAT® v9.4 and R v4.1 software.

### Role of the funding source

The funders had no role in study design, data collection, data analysis, interpretation, or writing of the report.

## Results

### Baseline characteristics of participants

Between 15th October 2018 and 18th January 2019, 1444 participants were enrolled in the RDS survey. Overall, 1039/1443 (72%) participants had positive HCV serology, and 875 (84%) of them had hepatitis C. The median age of the latter was 41 years (IQR: 36–47), 96% were males, 44% reported being on methadone treatment and 39% reported injecting drugs for 15 years or more (Table 1). Overall, 316 (36%) were HIV-coinfected, and among the HIV-infected participants, 77% had HIV-1 RNA ≤20 copies/mL and 49% had CD4-cell count >500 cells/μL. About half of all participants (54%) were not engaged in either addiction (methadone) or HIV care.

**Table 1:** Baseline characteristics of participants identified with hepatitis C.

	Treatment initiated (N = 686)	Treatment not initiated (N = 189)	p value^h^	Total (N = 875)
Median age of participants (IQR)	41 (37; 47)	38 (32; 45)	<0.01	41 (36–47)
Male gender, n (%)	662 (96.5)	179 (94.7)	0.26	841 (96.1)
Lived in couple, n (%)	311 (45.3)	66 (34.9)	0.01	377 (43.1)
Ever been homeless, n (%)	105 (15.3)	46 (24.3)	<0.01	151 (17.3)
Had a regular place to stay (last 6 months) n (%)	664 (96.8)	170 (90.0)	<0.01	834 (95.3)
Had a valid health insurance, n (%)	393 (57.3)	66 (34.9)	<0.01	459 (52.5)
Duration of heroin injection, n (%)			<0.01	
<5 years	31 (4.5)	22 (11.6)		53 (6.1)
5–<10 years	162 (23.6)	67 (35.5)		229 (26.2)
10–<15 years	207 (30.2)	46 (24.3)		253 (28.9)
≥15 years	286 (41.7)	54 (28.6)		340 (38.9)
Smoked methamphetamine in the last 30 days, n (%)	284 (41.4)	87 (46.3)^a^	0.23	371 (42.5)^a^
Non injecting drug use (last 6 months), n (%)	58 (8.5)^a^	24 (12.7)	0.08	82 (9.4)^a^
At risk consumption of alcohol (last 6 months)^f^, n (%)	192 (28.0)	57 (30.2)	0.56	249 (28.5)
Binge drinking (last 6 months)^g^, n (%)	55 (8.0)	16 (8.5)	0.84	71 (8.1)
Being on methadone treatment, n (%)	346 (50.4)	36 (19.2)^a^	<0.01	382 (43.7)^a^
HIV co-infection, n (%)	267 (38.9)	49 (25.9)	<0.01	316 (36.1)
*Among HIV positive participants (N* = *316)*				
HIV viral load ≤20 copies/mL, n (%)	216 (81.5)^b^	26 (53.1)	<0.01	242 (77.1)^b^
Being on ART, n (%)	237 (89.8)^c^	27 (57.5)^b^	<0.01	264 (84.9)^e^
CD4> 500 cells/mm^3^, n (%)	138 (51.7)	18 (36.7)	0.05	156 (49.4)
Engaged in HIV/addiction care, n (%)	419 (61.2)^a^	54 (29.0)^c^	<0.01	473 (54.3)^d^

Percentages may not total 100 because of rounding.

a One missing value.

b 2 missing value.

c 3 missing value.

d 4 missing value.

e 5 missing value.

f At risk consumption of alcohol when AUDITC score≥3 for women and AUDITC score ≥4 for Men.

g Binge drinking when frequency of drinking 5 or more drinks in one occasion is monthly or more.

h Based on Chi square tests for qualitative variables and median test for qualitative variables.

### HCV testing, treatment initiation and outcomes

**Fig. 1:**
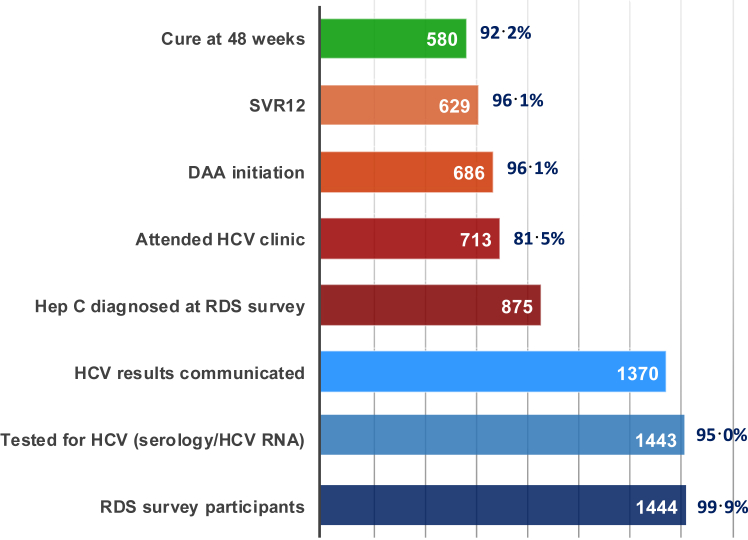
Cascade of hepatitis C testing and care among PWID in Hai Phong, Vietnam. The blue bars display the hepatitis C testing cascade. The orange bars display the hepatitis C care cascade.

**Fig. 2:**
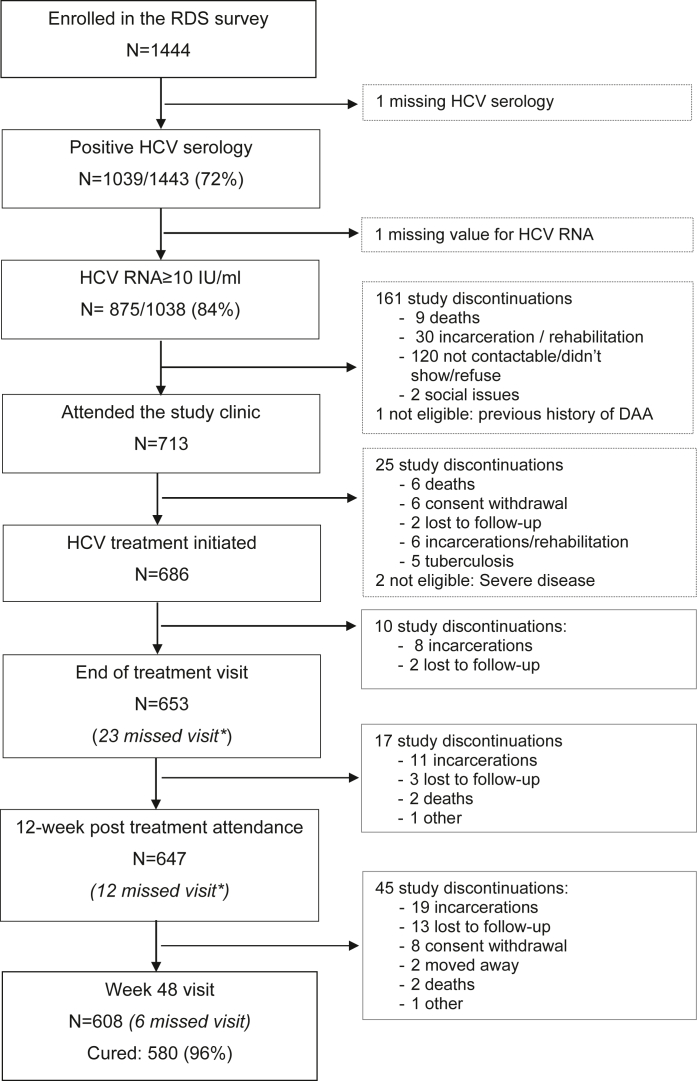
Participants enrolment and follow-up. ∗These participants have missed this visit but have attended at least one later visit.

Among the initial 1444 RDS survey participants, 99.9% were tested for HCV serology/HCV RNA, and the test results could be communicated to 95% of participants (Fig. 1). Among the 875 participants with hepatitis C, 713 (81%) attended one of the three clinical sites for further care and treatment within a maximum of 1 year (Fig. 2). Of them, 686/713 PWID (96%) were initiated on DAA. The main reasons for not initiating DAA were the inability to contact participants, non-attendance to the clinic, incarceration or retention in a rehabilitation centre and death (Fig. 2). The median time from the first eligibility visit at the clinic to treatment initiation was 8 days (IQR 7–21). Change in ART regimen, logistical and calendar constraints, late methadone initiation, or medical conditions (e.g., chronic hepatitis B, tuberculosis) delayed treatment initiation for some patients. Among those who initiated DAA, 111/686 (16%) had advanced liver fibrosis (F3/F4 stages), including 55 (8%) with cirrhosis. None had decompensated cirrhosis.

In the final multivariable model (Table 2), after initial selection of variables through a univariate analysis with a threshold of p < 0.15, the factors independently associated with not having initiated DAA following hepatitis C diagnosis at RDS were age <36 years (OR = 2.6, 95% CI: 1.6–4.4), not having a regular place to stay (OR = 2.4, 95% CI: 1.2–4.9), and not being engaged in either addiction or HIV care (OR = 2.8, 95% CI: 1.9–4.2).

**Table 2:** Factors associated with not initiating treatment (N = 869^a^).

	Univariate analysis			Adjusted analysis		
	OR	95% CI	p-value^e^	aOR	95% CI	p-value^e^
Female gender	1.6	[0.7; 3.3]	0.3			
Age class in years			<0.001			<0.001
<36	3.2	[2.0; 5.0]		2.6	[1.6; 4.4]	
[36; 41]	1.0	[0.6; 1.6]		1.1	[0.6; 2.0]	
[41; 47]	1.3	[0.8; 2.2]		1.6	[0.9; 2.6]	
≥47	Ref			Ref		
Being single^d^	1.5	[1.1; 2.1]	0.02			
No regular place to stay	3.2	[1.7; 6.2]	<0.001	2.4	[1.2; 4.9]	0.02
Not having a health insurance card	2.5	[1.8; 3.5]	<0.001	1.5	[1.0; 2.1]	0.06
Duration of injection in years			<0.001			0.11
<5 years	4.0	[2.1; 7.4]		2.0	[1.0; 3.9]	
5–<10 years	2.3	[1.5; 3.5]		1.4	[0.9; 2.2]	
10–<15 years	1.2	[0.8; 1.9]		1.0	[0.6; 1.5]	
≥15 years	Ref			Ref		
Frequency of heroin injection last 30 days:			<0.001			
Less than 30 injections	Ref					
Between 30 and 60 injections	1.6	[0.9; 2.9]				
At least 60 injections	2.5	[1.5; 4.2]				
Smoking methamphetamine in last 30 days	1.2	[0.9; 1.7]	0.3			
Frequency of methamphetamine smoking ice the last 30 days			0.4			
No ice smoking	Ref					
<10 times	1.2	[0.8; 1.7]				
≥10 times	1.4	[0.8; 2.2]				
Consumption of other non-injecting drugs	1.6	[1.0; 2.6]	0.07			
At risk consumption of alcohol^b^	1.1	[0.8; 1.6]	0.5			
Binge drinking^c^	1.1	[0.6; 1.9]	0.8			
Not on methadone treatment	4.2	[2.9; 6.4]	<0.001			
Being HIV negative	1.9	[1.3; 2.8]	<0.001			
Not engaged in HIV/addiction care	3.8	[2.7; 5.5]	<0.001	2.8	[1.9; 4.2]	<0.001

a 6 participants with missing data.

b At risk consumption of alcohol when AUDITC score ≥3 for women and AUDITC score ≥4 for men.

c Binge drinking when frequency of drinking 5 or more drinks in one occasion is monthly or more.

d Being single is defined to have a marital status: single, widowed, separated or divorced.

e Likelihood-ratio test.

During follow-up, 97% of patients who initiated DAA attended all drug supply visits. A tailored drug supply scheme, with more frequent drug supplies, was adopted for only 46 participants (7%). During treatment follow-up, 75 (11%) patients reported at least one missed dose. At the end of treatment, 42% of participants reported drug injection over the past month, and 78% reported being on MOUD.

### Clinical endpoints

Among the 686 patients who started DAA, 647 (94%) attended the SVR12 visit and 629 (97.2%) patients achieved SVR12. Assuming the 39 participants who did not attend this visit failed to achieve SVR12, the SVR12 rate was 91.7% (95% CI: 90–94) among all patients who initiated DAA. At 48 weeks, 581 (95.6%) participants who attended this visit had HCV RNA <10 IU/mL. The strategy success at 48 weeks was 66.4% (95% CI: 63–69) among those identified with hepatitis C at the RDS survey (Fig. 1). No participant had a severe adverse event related to DAA or ribavirin intake.

### Sub-group analysis

Among those not engaged in either HIV or addiction (methadone) care at the RDS survey (N = 398), 67% were initiated on treatment, 80% (95% CI: 75–85) of the latter were cured 12 weeks post-treatment. The strategy success at week 48 of those identified with hepatitis C at the RDS survey who were not engaged in either HIV or addiction (methadone) care was 53% (95% CI: 48–58).

### Reinfections

Among those with SVR12 who attended week 48 visit (N = 587, median follow-up 24 weeks, IQR: 23.7–24.3), 11 had detectable HCV RNA, yielding an HCV reinfection incidence of 4.1/100 person-years (95% CI: 2.0–7.3). This reinfection incidence was 3.7/100 person-years (95% CI: 1.6–7.4) among those on MOUD at SVR12, and 5.7/100 person-years (95% CI: 1.2–16.8) among those not on MOUD.

## Discussion

In a LMIC country, our strategy allowed screening large numbers of ‘current’ PWID recruited from the community in a short period (about 1500 in 3 months), curing and preventing reinfection for two thirds of those identified with hepatitis C. Of note, this success rate remained high among those not initially engaged in any HIV or addiction care.

To the best of our knowledge, this is the first study testing a comprehensive HCV micro-elimination strategy in a LMIC among PWID. Previous studies have addressed only some components of the screening, care and prevention pathway, and most enrolled limited numbers of PWID. The CT2 study in Myanmar reported high cure rates (SVR12) among PWID attending a drop-in centre.^9^ In Iran, a community-based study enrolled 134 patients identified with hepatitis C from drop-in centres or methadone clinics with 84% treatment initiation, but the screening and cure rates were not reported.^8^ In Kenya, among 95 PWID attending a MOUD and needle-syringe program facility, 79 (85.9%) were cured at 12 weeks post treatment by ledipasvir/sofosbuvir.^24^ In Georgia, among the 2600 PWID attending a needle/syringe program, the testing rate was only 21%. The PCR RNA uptake was not reported in this study, but 74% of those positive (N = 244) were initiated on DAA with a cure rate of 85%.^25^ The largest reported test & treat program has been implemented in India, focusing on PWID attending health care services. In 25 local hospitals over three years, 3477 PWID were identified with HCV RNA and treated. However, the screening rate was not reported. Among those treated, the SVR12 rate was only 40%.^6^ Our community-based strategy, encompassing HCV testing, treatment and reinfection, clearly outperforms these previous reports in terms of screening and cure rates. Interestingly, while HCV incidence was estimated at 19.4/100 person-years (95% CI: 11.5–30.7) among PWID a few years before this study in the same setting,^21^ reflecting a high HCV transmission level in this population, the reinfection rate (4.1/100 person-years), though slightly higher than reported from upper-middle income countries (2.8/100 person-years in a meta-analysis^26^), is reassuring.

We believe that several factors have been pivotal to this success. Among them, the involvement of CBO during the study conception, RDS survey implementation and all stages of the cascade of care, including their presence in the HCV care clinics, was crucial. In particular, the were key in reaching high follow-up, high cure rate through adherence support, and a very high rate of PWID on MOUD (78%) at the end of their DAA treatment. Finally, their provision of harm reduction and HCV prevention also likely contributed to reduce the HCV reinfection rate.^27^ In addition to this major CBO involvement, undertaking HCV screening and diagnosis within the community level enabled a high coverage of screening by avoiding referral to the health system for diagnosis purposes which can lead to significant loss to follow-up.^28^ Conversely, given the tailored drug supply scheme was actually useful for only 7% of participants, it likely did not make a difference in that context, but it could be useful in other countries.

Although successful, our strategy could be improved in future initiatives by allocating more CBO resources for counselling and follow-up among those PWID at high risk of not initiating DAA: the youngest PWID, those injecting for only few years only, those with no regular housing, and those not engaged in any care.

Our decentralised approach integrating HCV care into public services adheres to and strongly supports the recent WHO recommendations towards HCV elimination.^11^ Overall, our findings suggest this initiative could form the basis of a strategy to eliminate HCV among PWID. Indeed, we previously showed that repeating RDS surveys in the same targeted population (at the scale of a large city such as Hai Phong for example) allowed reaching up to two-thirds of this population in a relatively short period of time. An ongoing modelling exercise is currently assessing whether repeating the same screening, care and prevention pathway among PWID in Hai Phong, assuming a stable rate of HCV cure and reinfection, would be cost-effective and would allow reaching HCV micro-elimination. Given that HCV testing and treatment of PWID can also be implemented in existing health services such as methadone and HIV clinics, our findings strongly suggest that our strategy could fill the gap of reaching, testing, curing and preventing HCV reinfection among PWID from the community not currently engaged in care. The success rate among the latter, from HCV screening acceptance to reinfection at 48 weeks, remains high for this population but certainly could be improved. Potential solutions include a previous engagement with regular CBO services, building self-confidence and managing mental health comorbidities.^29^ This projection can only be envisaged through a wide access to DAA for PWID. The lowering cost of generic DAA for LMIC is changing the scene. In Vietnam, the expected integration of DAA among the medications covered by the national Health insurance should be a promising step forward. This wide access to DAA will need to be accompanied by a specific training of physician who may still be reluctant to treat PWID, or only those on MOUD or with cirrhosis, because of presumed high failure or reinfection rates.^3^ Of note, as more PWID will be treated in the target population, the reinfection risk should decrease further. Finally, our micro-elimination strategy could benefit from funds allocated by the Global Fund. In addition, our upcoming cost-effectiveness analysis will likely show that investing in such strategies through their reimbursement by the health insurance would save money in the future.

Our study has some limitations. It was implemented in a single setting and the findings may be difficult to generalizable to other locations. Interestingly though, our group has shown that a similar strategy could also be successful in a high-income country such as France where HCV care is free of charge for several years.^30^ We did not have a control group to compare the rate of HCV testing, cure and reinfection under the local standards of care for HCV (set for the general population). However, due to their costs, DAA remained inaccessible to PWID during this period. Through the DRIVE project, which started 3 years before, CBO involved in the study were experienced with RDS surveys and PWID case management for risk reduction counselling and support for attending methadone and HIV outpatient clinics. In other settings, less experimented CBO may be less efficient. Providing training and supervision could easily empower CBO with the required skills to play their key roles in similar study. Finally, the liver stiffness assessment, still a prerequisite to inform HCV treatment modalities, relied partially on portable Fibroscan® which may not be available everywhere. Additional analyses are ongoing to assess whether the APRI score alone could be used to identify patients with cirrhosis.

Although our findings should be confirmed in other settings, our strategy fills an important gap on the road to achieving HCV elimination among PWID.

## Contributors

N.N., K.P.M, V.H.V, O.T.H.K. P.V, L.W, M.L., K.L., and D.L. conceived and designed the study.

K.M.P., C.Q. D.T.H., D.L., J.P.M., and D.R. supervised the fieldwork.

R.V. and C.M.D. coordinated the data entry.

R.V., N.N., P.V.D.P., J.F., L.M., J.C. D.D.J., and D.L. contributed to the design of the data analysis.

R.V. and N.N. conducted the data and statistical analyses.

N.T.B, V.H.V., N.T.T.T, T.T.H, B.T.N.Q. implemented the fieldwork.

N.N and D.L wrote the manuscript which was then reviewed and approved by all authors.

## Data sharing statement

De-identified participant data and a data dictionary will be made available on request addressed to the corresponding author.

## Declaration of interests

ML received fees from Cepheid and Gilead US, as well as consultancy fees from Gilead US, outside the submitted work. KL received grants or contracts from MSD, honoraria from Janssen and Gilead, and travel support from Gilead, outside the submitted work. DDJ received grants and contracts from U.S. Centers for Disease Control, outside the submitted work. PV grants and contracts from Gilead medical Sciences outside the submitted work.

Other authors declare no competing interest.

## Appendix A Supplementary data

Appendix A - DRIVE-C Study group members

## References

[1] World Health Organization (2016).

[2] Degenhardt L., Peacock A., Colledge S. (2017). Global prevalence of injecting drug use and sociodemographic characteristics and prevalence of HIV, HBV, and HCV in people who inject drugs: a multistage systematic review. Lancet Glob Health.

[3] Cooke G.S., Andrieux-Meyer I., Applegate T.L. (2019). Accelerating the elimination of viral hepatitis: a lancet Gastroenterology & Hepatology commission. Lancet Gastroenterol Hepatol.

[4] Grebely J., Dalgard O., Conway B. (2018). Sofosbuvir and velpatasvir for hepatitis C virus infection in people with recent injection drug use (SIMPLIFY): an open-label, single-arm, phase 4, multicentre trial. Lancet Gastroenterol Hepatol.

[5] Martin N.K., Vickerman P., Dore G.J., Hickman M. (2015). The hepatitis C virus epidemics in key populations (including people who inject drugs, prisoners and MSM): the use of direct-acting antivirals as treatment for prevention. Curr Opin HIV AIDS.

[6] Dhiman R.K., Grover G.S., Premkumar M. (2021). Outcomes of real-world integrated HCV microelimination for people who inject drugs: an expansion of the Punjab model. eClinicalMedicine.

[7] Solomon S.S., Quinn T.C., Solomon S. (2020). Integrating HCV testing with HIV programs improves hepatitis C outcomes in people who inject drugs: a cluster-randomized trial. J Hepatol.

[8] Alavi M., Poustchi H., Merat S. (2019). An intervention to improve HCV testing, linkage to care, and treatment among people who use drugs in Tehran, Iran: the ENHANCE study. Int J Drug Policy.

[9] Draper B.L., Htay H., Pedrana A. (2021). Outcomes of the CT2 study: a ‘one-stop-shop’ for community-based hepatitis C testing and treatment in Yangon, Myanmar. Liver Int.

[10] (2017). Global hepatitis report. https://www.who.int/publications-detail-redirect/global-hepatitis-report-2017.

[11] Updated recommendations on simplified service delivery and diagnostics for hepatitis C infection. https://www.who.int/publications-detail-redirect/9789240052697.

[12] Day E., Hellard M., Treloar C. (2019). Hepatitis C elimination among people who inject drugs: challenges and recommendations for action within a health systems framework. Liver Int.

[13] Grebely J., Hajarizadeh B., Lazarus J.V., Bruneau J., Treloar C. (2019). International network on hepatitis in substance users. Elimination of hepatitis C virus infection among people who use drugs: ensuring equitable access to prevention, treatment, and care for all. Int J Drug Policy.

[14] Duong H.T., Moles J.P., Pham K.M. (2022). A community-based intervention to decrease the prevalence of HIV viremia among people who inject drugs in Vietnam. Lancet Reg Health West Pac.

[15] Sypsa V., Psichogiou M., Paraskevis D. (2017). Rapid decline in HIV incidence among persons who inject drugs during a fast-track combination prevention program after an HIV outbreak in Athens. J Infect Dis.

[16] Solomon S.S., McFall A.M., Lucas G.M. (2017). Respondent-driven sampling for identification of HIV- and HCV-infected people who inject drugs and men who have sex with men in India: a cross-sectional, community-based analysis. PLoS Med.

[17] Falade-Nwulia O., Ward K.M., McCormick S. (2020). Network-based recruitment of people who inject drugs for hepatitis C testing and linkage to care. J Viral Hepat.

[18] Kikvidze T., Luhmann N., Avril E. (2018). Harm reduction-based and peer-supported hepatitis C treatment for people who inject drugs in Georgia. Int J Drug Policy.

[19] Des Jarlais D.C., Huong D.T., Oanh K.T.H. (2020). Ending an HIV epidemic among persons who inject drugs in a middle-income country: extremely low HIV incidence among persons who inject drugs in Hai Phong, Viet Nam. AIDS Lond Engl.

[20] Zhang L., Celentano D.D., Le Minh N. (2015). Prevalence and correlates of HCV monoinfection and HIV and HCV coinfection among persons who inject drugs in Vietnam. Eur J Gastroenterol Hepatol.

[21] Molès J.P., Vallo R., Khue P.M. (2020). HIV control programs reduce HIV incidence but not HCV incidence among people who inject drugs in HaiPhong, Vietnam. Sci Rep.

[22] Rapoud D., Quillet C., Pham Minh K. (2020). Towards HCV elimination among people who inject drugs in Hai Phong, Vietnam: study protocol for an effectiveness-implementation trial evaluating an integrated model of HCV care (DRIVE-C: DRug use & Infections in ViEtnam-hepatitis C). BMJ Open.

[23] Lan C.W., Lin C., Thanh D.C., Li L. (2018). Drug-related stigma and access to care among people who inject drugs in Vietnam. Drug Alcohol Rev.

[24] Akiyama M.J., Riback L.R., Nyakowa M. (2022). Hepatitis C treatment outcomes among people who inject drugs accessing harm reduction settings in Kenya. J Viral Hepat.

[25] Bouscaillou J., Kikvidze T., Butsashvili M. (2018). Direct acting antiviral-based treatment of hepatitis C virus infection among people who inject drugs in Georgia: a prospective cohort study. Int J Drug Policy.

[26] Muller A., Vlahov D., Akiyama M.J., Kurth A. (2020). Hepatitis C reinfection in people who inject drugs in resource-limited countries: a systematic review and analysis. Int J Environ Res Public Health.

[27] Hajarizadeh B., Cunningham E.B., Valerio H. (2020). Hepatitis C reinfection after successful antiviral treatment among people who inject drugs: a meta-analysis. J Hepatol.

[28] Nagot N., Hai V.V., Dong T.T.T. (2022). Alarming tuberculosis rate among people who inject drugs in Vietnam. Open Forum Infect Dis.

[29] Michel L., Le S.M., Thi G.H. (2022). Assessment of a psychiatric intervention at community level for people who inject drugs in a low-middle income country: the DRIVE-Mind cohort study in Hai Phong, Viet Nam. Lancet Reg Health West Pac.

[30] Nagot N., D'Ottavi M., Quillet C. (2022). Reaching hard-to-reach people who use drugs: a community-based strategy for the elimination of hepatitis C. Open Forum Infect Dis.

