# Supplementary material for: A community-based strategy to eliminate hepatitis C among people who inject drugs in Vietnam
Source: Lancet Reg Health West Pac. 2023 May 27;37:100801. doi: 10.1016/j.lanwpc.2023.100801 (PMC10485672; doi:10.1016/j.lanwpc.2023.100801)
Supplement: Appendix A - DRIVE-C Study group members [file mmc1.docx]

**Appendix A** - DRIVE-C Study group members

**International coordination centre:** Didier Laureillard, Nicolas Nagot, Don Des Jarlais, Jonathan Feelemyer, Catherine Quillet, Roselyne Vallo, Jean-Pierre Moles, Laurence Weiss, Maud Lemoine, Laurent Michel.

**Hai Phong University of Medicine and Pharmacy coordination centre:** Pham Minh Khue, Delphine Rapoud, Nguyen Thanh Binh, Tran Thi Hong, Nguyen Thi Thanh Hang, Phung Quang Hai, Vu Thi Thom, Cap Minh Duc, Le Thi Thuy Linh, Bach Thi Nhu Quynh, Le Thuy Linh, Nguyen Thi hong, Vu Thi Thu Trang.

**Viet Tiep hospital:** Vu Hai Vinh, Nguyen Thi Thanh Huong, Hoang Thi Thia, Nguyen Thi Van Anh, Vu Thi Sinh, Doan Thi Hai Binh.

**Thuy Nguyen hospital:** Nguyen Van Luc, Vu Thi Thu Ha, Do Thi Nhung, Bui Minh Khoi, Bui Thi Thien, La Thi Vu Quang, Le Huu Toi, Hoang Thi Luong, Pham Thi Thanh Phuong.

**Kien An hospital:**Pham Thi Lieu, Pham Thi Thanh Mai, Trinh Thi Hoa, Bui Thi Thu Trang, Nguyen Thi Thu Ha, Nguyen Thi Xuyen, Trinh Thi Huong, Nguyen Thi Minh, Phung Thi Thuy, Tran Thị Duyen, Vu Thi Que.

**Community based organizations:** Nguyen Duc Dung, Nguyen Duc Tuan, Nguyen Hoang Long, Nguyen Manh Hung, Vu Van Tu, Nguyen Thanh Kien, Pham Thi Thu Hong, Truong Thi Cuc, Nguyen Minh Quan, Nguyen Thi Lan, Nguyen Thi Va, Nguyen The Manh, Doan Van Cuong, Pham Van Hoan, Cao Thi Kim Giang, Ha Quang Hiep, Ngo Trong Ninh, Nguyen Anh Quan, Vu Thi Bich Hop, Nguyen Thi Thu, Nguyen Thi Sau, Hoang Van Tuan, Nguyen Huu Nhan, Nguyen Quoc Tuan, Le Tuan Long, Vu Minh Son, Tran Viet Hoc, Nguyen Van Dinh, Nguyen Hoang, Pham Thi Hung, Trinh Thai Binh, Le Thi Hang, Hoang Dang Quan, Nguyen Thị Thanh, Le Thi Loan, La Cao Cuong, Tran Van Ha, Tran Van Thao, Nguyen Xuan Truong, Nguyen Duc Hanh, Nguyen Thi Loan, Tran Lam Tung, Hoang Van Tuan, Tran Thi Lien.

**SCDI coordination:**Khuat Thi Hai Oanh, Nham Thi Tuyet Thanh, Bui Thi Thuy Linh, Ngo Thi Dien.

**Modelling and cost**

Peter Vickerman, Hannah Fraser, Josephine Walker, Adam Trickey
